# Functional Neuroanatomy of Executive Function after Neonatal Brain Injury in Adults Who Were Born Very Preterm

**DOI:** 10.1371/journal.pone.0113975

**Published:** 2014-12-01

**Authors:** Anastasia K. Kalpakidou, Matthew P. G. Allin, Muriel Walshe, Vincent Giampietro, Philip K. McGuire, Larry Rifkin, Robin M. Murray, Chiara Nosarti

**Affiliations:** 1 Department of Psychosis Studies, Institute of Psychiatry, King's Health Partners, King's College London, London, United Kingdom; 2 Department of Neuroimaging, Institute of Psychiatry, King's Health Partners, King's College London, London, United Kingdom; Hangzhou Normal University, China

## Abstract

Individuals who were born very preterm (VPT; <33 gestational weeks) are at risk of experiencing deficits in tasks involving executive function in childhood and beyond. In addition, the type and severity of neonatal brain injury associated with very preterm birth may exert differential effects on executive functioning by altering its neuroanatomical substrates. Here we addressed this question by investigating with functional magnetic resonance imaging (fMRI) the haemodynamic response during executive-type processing using a phonological verbal fluency and a working memory task in VPT-born young adults who had experienced differing degrees of neonatal brain injury. 12 VPT individuals with a history of periventricular haemorrhage and ventricular dilatation (PVH+VD), 17 VPT individuals with a history of uncomplicated periventricular haemorrhage (UPVH), 13 VPT individuals with no history of neonatal brain injury and 17 controls received an MRI scan whilst completing a verbal fluency task with two cognitive loads (‘easy’ and ‘hard’ letters). Two groups of VPT individuals (PVH+VD; n = 10, UPVH; n = 8) performed an n-back task with three cognitive loads (1-, 2-, 3-back). Results demonstrated that VPT individuals displayed hyperactivation in frontal, temporal, and parietal cortices and in caudate nucleus, insula and thalamus compared to controls, as demands of the verbal fluency task increased, regardless of type of neonatal brain injury. On the other hand, during the n-back task and as working memory load increased, the PVH+VD group showed less engagement of the frontal cortex than the UPVH group. In conclusion, this study suggests that the functional neuroanatomy of different executive-type processes is altered following VPT birth and that neural activation associated with specific aspects of executive function (i.e., working memory) may be particularly sensitive to the extent of neonatal brain injury.

## Introduction

‘Executive function’ in the neuropsychological literature refers to a variety of cognitive operations that permit the adaptive balance of maintenance and shifting of cognitive and behavioural responses to environmental demands, allowing the control of action and long-term goal-directed behavior [1]. These include inhibitory control, attention allocation, task initiation, working memory, mental flexibility, planning and problem-solving [2]. Individuals who were born very preterm (VPT; <33 gestational weeks) are at increased risk of experiencing impairments in executive function [3], especially those with a history of neonatal brain injury [4], [5].

Periventricular haemorrhage (PVH) is well-recognized on cranial neonatal ultrasounds and occurs in up to a quarter of VPT infants [6]. It originates in the subependymal germinal matrix, a transient metabolically-rich structure of the developing brain proliferating neuronal and glial precursor cells, that disappears by term. Anatomically, the germinal matrix lies predominantly adjacent to the head of the caudate nucleus during the last trimester of gestation [7]. PVH has been associated with impaired cortical growth in VPT infants [8], as well as with structural and functional alterations in caudate nucleus in VPT individuals in adolescence [9], [10]. Therefore, PVH may lead to alterations in areas subserving the cognitive operations involved in executive function processing by disrupting the development of fronto-striatal circuits [11] and specifically the connections between the caudate nucleus, the hippocampus and the frontal and parietal association cortices [12], [13].

PVH may occur either in isolation (i.e., uncomplicated periventricular haemorrhage-UPVH) or may be associated with ventricular dilatation (VD; PVH+VD) following extension of the haemorrhage in the lateral ventricles [14], [15]. PVH+VD is likely to cause the greatest alterations in grey and white matter volumes in VPT individuals [16], with increased ventricular volume being associated with regional volume loss in brain areas in key nodes of the ‘executive’ network, i.e., hippocampus, caudate nucleus and frontal and parietal cortices [17].

In terms of long-term functional brain alterations following neonatal brain injury, we recently demonstrated that frontal and parietal blood-oxygen-level dependent (BOLD) signal linearly decreased with increasing neonatal ultrasound abnormalities in VPT young adults during completion of a verbal paired associates learning task [18]. To the best of our knowledge, the effect of differing degrees of neonatal brain injury on the functional neuroanatomy of executive function among VPT individuals has not been previously studied.

In this paper we focus on two cognitive tasks, which tap different aspects of executive processing. The first is phonological verbal fluency, which involves processes such as attention allocation, response initiation, response monitoring and working memory [19], [20] and is mainly subserved by frontal and striatal brain regions [21], [22]. Individuals who were born VPT typically score approximately half a standard deviation below controls' scores on this type of task [23]. We previously studied the functional neuroanatomy of phonological verbal fluency in a VPT-born adult heterogeneous sample (with no history of neonatal PVH+VD), using an fMRI task with differing cognitive loads (‘easy’, ‘hard’ letters) [24]. In our previous study, we reported differential patterns of brain activation in a fronto-striatal neural network between VPT individuals and controls. In the current study, we investigate the haemodynamic response to the same verbal fluency task in three groups of VPT young adults with the following neonatal ultrasound classifications: 1) PVH+VD 2) UPVH and 3) normal ultrasographic findings, and a group of term-born controls.

The second task is an n-back task, which assesses working memory. N-back paradigms typically engage neural networks that subserve processes of executive control of verbal encoding and retrieval and active maintenance processes i.e., frontal and parietal brain regions, respectively [25], [26]. At the behavioural level, significant group differences of typically 0.4 standard deviation are observed in VPT samples in favour of term-born controls [23]. Only a few fMRI studies of working memory with VPT samples have been conducted to date and have reported significant neuroanatomical differences between VPT individuals and controls. A pioneering study of spatial working memory by Curtis and co-workers (2006) [27] showed decreased activation in the caudate nucleus of early adolescents who were born very preterm compared to controls. Furthermore, a recent investigation by Griffiths and colleagues (2013) [28], employing a selective attention/working memory task, demonstrated decreased activation in a working memory network comprising fronto-parietal cortices, as well as in occipital areas, in children born extremely preterm (<28 gestational weeks) compared to controls. Here, we used a verbal n-back task with differential working memory loads (1-, 2-, 3-back) in two groups of VPT individuals: 1) PVH+VD and 2) UPVH.

We hypothesise that there would be differential activation in VPT individuals with differing degrees of neonatal brain injury [18] in selective components of the ‘executive’ network, specifically, in caudate, dorsolateral prefrontal cortex, superior frontal and medial parietal brain regions, especially as the cognitive load of the tasks increased [29]–[31].

## Materials and Methods

### Participants

VPT participants were drawn from a cohort of 368 individuals who were born before 33 gestational weeks in 1979–1984. All individuals were admitted to the Neonatal Unit at the University College London Hospital, where they received neonatal ultrasound scans daily for the first 4 days of life, at 1 week, and weekly until they were discharged from hospital [14]. These infants were all enrolled for participation in longitudinal follow-up studies [32]–[34]. At 14–15 years, 269 individuals of the original cohort agreed to be assessed. Results of the adolescent assessment have been previously published [35]–[37]. At 19–20 years, 94 individuals of those assessed in adolescence underwent further neuropsychological assessment [38]. Out of 94 individuals, 87 agreed to receive structural MRI scan as well [39]. A sub-sample of these individuals participated in a series of fMRI studies [24], [40]–[42]. The current study included 42 VPT individuals; 22 VPT individuals who had previously participated in fMRI studies (mean age at assessment: 20.28 years) [24], [40]–[42] and 20 newly-recruited VPT individuals i.e., they had not been involved in the fMRI studies (mean age at assessment: 25.2 years), who had been part of the original cohort. The VPT individuals who participated in previous fMRI studies did not significantly differ from those VPT individuals who did not participate in these studies (but were assessed at 19–20 years) in full-scale intelligence quotient (IQ), as measured with Wechsler Abbreviated Scale of Intelligence (WASI) [43] (z = −1.09, p = 0.28). All participants were chosen on the basis of their neonatal ultrasonographic findings i.e., normal results (normal VPT, n = 13; 11 previously studied, 2 newly-recruited), UPVH (n = 17; 9 previously studied, 8 newly-recruited), and PVH+VD (n = 12; 2 previously studied, 10 newly-recruited) [18], [36]. All VPT individuals performed a phonological verbal fluency task, while only newly-recruited VPT individuals (UPVH, n = 8; PVH+VD, n = 10) completed a working memory n-back task. Exclusion criteria were: severe head injury, stroke, epilepsy, multiple sclerosis, severe eyesight impairment, hearing and/or motor impairment, and pregnancy for female participants. Researchers were not blind to neonatal ultrasonographic findings at time of assessment. All data analyses were performed blind to group membership up until group level statistics.

Term-born control data (37–42 gestational weeks, n = 17, mean age at assessment: 20.75 years) were available for the verbal fluency task [24]. Exclusion criteria, other than those common to the VPT participants, were: birth complications (e.g., low birth weight defined as <2500 grams, endotracheal mechanical ventilation) and history of psychiatric illness.

Ethical approval was granted by the local ethical committee i.e., the Institute of Psychiatry Research Ethics Committee (reference number: 149/02) and King's College London Ethics Committee (reference number: 06/Q0703/97). Written informed consent was acquired by all participants, who were adults with capacity to provide informed consent. Consent documentation procedure was approved by the above ethical committees.

All participants were dextral, English native speakers.

### Neonatal, socio-demographic and neuropsychological data

Neonatal data i.e., birth weight (grams) and gestational age at birth (weeks) were collected for VPT study participants only. Information about sex, age at assessment, and socio-economic status (SES) [44] was available for all study participants.

Four subtests from theWASI [43] (vocabulary, block design, similarities and matrix reasoning) were used to estimate verbal, performance and full-scale IQ.

A measure of executive function, the Stockings of Cambridge test (SoC), from the Cambridge Neuropsychological Test Automated Battery (CANTAB) (CANTABeclipse version, 2003), focusing on problem solving, was administered on VPT individuals who performed the n-back task.

### fMRI Tasks

#### Verbal Fluency

All participants completed a phonological verbal fluency task, which we have previously used [24]. They were instructed to overtly generate a word beginning with the letter presented on the screen, avoiding proper names and repetitions and grammatical variations of a previous word [45]. On failure to generate a word, participants were asked to articulate the word ‘pass’.

The task was made up of two conditions (‘hard’, ‘easy’) and a baseline (‘rest’), which were presented in a total of 15, 35-second blocks. Each block consisted of 7 consecutive presentations of a given letter-stimulus (task conditions) or the word ‘rest’ (baseline) with a 5 s inter-stimulus interval (ISI). Each condition and the baseline were repeated five times. The ‘hard’ and the ‘easy’ conditions involved the following set of letters, respectively: I, F, O, N, E and C, P, S, T, L. Letter selection was made on the basis of sufficient power provided by this number of stimuli for detecting regional brain activation [46], [47]. Stimuli were divided into ‘easy’ and ‘hard’ letters [24], according to the frequency of English words beginning with those letters [48]. High frequency letters (‘easy’) evoke a larger number of automated responses compared to low frequency letters (‘difficult’) and are therefore more discriminative in group comparisons [48].

The order of letter presentation was reversed for alternate participants. During baseline, participants were asked to read the word ‘rest’ aloud. Verbal responses were recorded using an MRI-compatible microphone using Cool Edit 2000 (Syntrillium Software Corporation).

#### N-back

We used an n-back task with three conditions (1-, 2- 3-back) and a baseline (0-back). These were presented in a total of 18, 28-second blocks, each consisting of 14 consecutive presentations of letters [49]. Each condition was alternated with the baseline, which was always presented first (3 and 9 repetitions, respectively). Participants were presented with a series of letters, one at a time, at the centre of the screen, with a 2 s ISI, and were asked to press a button whenever the presented letter matched the one presented ‘n’ trials before (1-, 2-, and 3-back). In the baseline (0-back), participants were instructed to press a button whenever they saw the letter ‘X’ on the screen.

Task performance was recorded on-line and individual scores were calculated using a signal detection (*d′* prime) measure, which takes into account correct responses and false alarms [50]. *d′* prime is calculated as: Z(hit rate) - Z(false alarm rate). Reaction times were also recorded on-line.

### Image acquisition

Scans were performed using a 1.5 Tesla GE MR Signa System at the Maudsley Hospital, London. T2*-weighted functional volumes were acquired (repetition time-TR = 2000 ms, echo-time-TE = 40 ms, flip angle = 90^o^, in-plane resolution = 3.75^2^) in 22 axial slices (slice thickness = 5 mm, gap = 0.5 mm) for the verbal fluency task (109 volumes) and, in 16 axial slices (slice thickness = 7 mm, gap = 0.7 mm) for the n-back task (270 volumes). A 43-slice high-resolution gradient echo structural image was also collected (slice thickness = 3 mm, gap = 0.3 mm, TR = 3000 ms, TE = 40 ms, flip angle = 90^0^, in-plane resolution = 1.88^2^) and was used to normalize the individual functional data into standard space.

### fMRI data analysis

The data were analysed using the XBAM (version 4.1) software, developed at King's College London, Institute of Psychiatry, which uses a non-parametric approach based on permutation strategies (for a full description and references see http://www.brainmap.co.uk). Following motion correction and smoothing with a Gaussian filter (FWHM 8.8 mm), single subject analyses in native space were performed.

The estimated BOLD effect was modelled using two Gamma variate functions and the sum of squares (SSQ) ratio, a goodness-of-fit statistic, was computed at each voxel. The data were then permuted and individual brain activation maps for each task condition were created [51]. To reduce the possible confounding effects of differential task performance on BOLD signal, only activations related to correct responses were considered for both verbal fluency and n-back tasks.

Individual brain activation maps were then transformed into a standard Talairach space [52]. Group brain activation maps were then computed for each task condition using the median of the SSQ ratio over all individuals at each voxel and comparing them to those obtained from repeating the process with the permuted (null) data. The analysis was then extended from the voxel to the 3D cluster level.

Comparisons of responses across groups and task conditions were conducted by fitting the data at each intracerebral voxel at which all individuals had non-zero data. The null distribution was computed by permuting data across groups/conditions numerous times, under the assumption of no condition or group effect, followed by refitting of the above model.

For the *verbal fluency task*, we used a monotonic trend to compare the four groups (PVH+VD, UPVH, normal VPT, controls) across conditions (‘easy’, ‘hard’). This was a 4 (group) x 2 (task condition) factorial analysis of variance (ANOVA). Age at assessment was used as a covariate in the analyses as participants' age statistically differed between the groups (see Table 1).

**Table 1:** Neonatal, socio-demographic, neuropsychological and on-line behavioural data of the study groups performing a verbal fluency task.

Variable +	PVH+VD (n = 12)	UPVH (n = 17)	Normal VPT (n = 13)	Controls (n = 17)	Statistics
Neonatal/socio-demographic characteristics					
• Birth-weight (grams)	1122.33 (395.6)	1274.47 (396.7)	1347.38 (404.15)	n/a ^++^	F_(2,39)_ = 1.03, p = 0.37
• Gestation at birth (weeks)	28.42 (2.64)	28.76 (2.14)	29.38 (2.29)	n/a	F_(2,39)_ = 0.56, p = 0.58
• Males/Females (number)	7/5	8/9	6/7	8/9	x^2^_(3)_ = 0.51, p = 0.92
• Age (yrs) at assessment *	24.58 (2.48)	22.65 (2.57)	20.80 (1.8)	20.75 (1.37)	F_(3,55)_ = 9.99, p<0.001
• SES at assessment (number) ^a^					x^2^_(6)_ = 6.21, p = 0.4
I–II	6	10	4	7	
III	6	5	7	5	
IV–V	0	2	2	4	
Neuropsychological performance (WASI) ^b^					
• Full-scale IQ	105.92 (6.97)	106.47 (9.77)	96.46 (12.43)	107.71 (13.93)	F_(3,51)_ = 2.53, p = 0.07
• Verbal IQ	104.75 (10.07)	102.06 (9.62)	94.15 (11.79)	105.29 (12.12)	F_(3,51)_ = 2.5, p = 0.07
• Performance IQ	106.08 (11.41)	109.06 (10.63)	99.23 (12.89)	108.43 (15)	F_(3,51)_ = 1.61, p = 0.2
On-line task performance: Accuracy (% correct responses) ^c^					
• Easy Condition	83.37 (12.17)	90.09 (9.49)	84.83 (12.27)	91.09 (7.89)	F_(3, 54)_ = 1.89, p = 0.14
• Hard Condition	70.66 (14.29)	75.11 (14.63)	66.4 (15.77)	78.31 (10.17)	F_(3, 54)_ = 1.79, p = 0.16

+ Mean and standard deviation (SD) are presented, unless otherwise stated ^++^ n/a = non-applicable; neonatal data were not available for controls *p<0.001, Post-hoc comparisons with a Games-Howell test showed that the PVH+VD group was significantly older than the normal VPT [Mean difference; MD = 3.78, p<0.005] and the control groups [MD = 3.83, p<0.005] ^a^ For controls n = 1 missing data ^b^ For controls n = 3 missing data; age at assessment was used as a covariate ^c^ For PVH+VD, n = 11; 1 participant was excluded from fMRI data analysis due to problems with scan acquisition.

For the *n-back task*, we explored the interaction of group (PVH+VD, UPVH) and task condition (working memory load) using 2 (group) x 3 (task condition) factorial ANOVA. Given that there were no significant BOLD signal differences between the 2- and 3-back conditions within each group, a 2 (group) x 2 (task condition; 1-, 3-back) factorial ANOVA was performed.

For both tasks, resulting maps were statistically thresholded in such a way as to yield less than 1 false positive 3D cluster per map. SSQ values were extracted from cluster mean where significant interaction effects were observed, in order to be used for graphical representation of the data in the results section.

### Statistical analysis of non-imaging data

Statistical analyses were carried out with SPSS v20.0 (Chigago, USA). To explore possible between-group differences in sex and SES [44] distribution, a Chi-square test for independence (x^2^) was used. Between-group comparisons in terms of age at assessment, neonatal, neuropsychological [43] and on-line behavioural data were performed using one-way univariate ANOVA for comparison of 3 or more groups, or a student's *t* test or a Mann-Whitney test, according to data distribution, for comparisons of 2 groups.

To explore the link between task performance and fMRI data, correlation analysis was performed between SSQ values extracted from brain regions where significant interaction effects were observed and measures of task performance.

## Results

### Sample characteristics

Table 1 summarises neonatal, socio-demographic and neuropsychological data for groups of participants that performed the verbal fluency task. Groups significantly differed in age at assessment. There were no significant between-group differences in IQ.

Study groups that performed the n-back task did not significantly differ in neonatal, socio-demographic and neuropsychological data (Table 2).

**Table 2:** Neonatal, socio-demographic, neuropsychological and on-line behavioural data of the PVH+VD and UPVH groups performing an n-back task.

Variable +	PVH+VD (n = 10) (n = 13)	UPVH (n = 8)	Statistics
Neonatal/socio-demographic characteristics			
• Birth-weight (grams)	1150.6 (380.86)	1208 (322.75)	F_(16)_ = 0.35, p = 0.74
• Gestation at birth (weeks)	28.7 (2.45)	28.75 (2.44)	F_(16)_ = 0.003, p = 0.97
• Males/Females (number)	6/4	2/6	x^2^_(1)_ = 1.02, p = 0.31
• Age (yrs) at assessment	25.4 (1.71)	25.13 (1.25)	F _(16)_ = 1.2, p = 0.71
• SES at assessment (number)			x^2^_(2)_ = 2.61, p = 0.27
I–II	6	6	
III	4	1	
IV–V	0	1	
Neuropsychological performance			
• WASI			
Full-scale IQ	106.7 (7.42)	104.25 (11.34)	F_(16)_ = 1.52, p = 0.59
Verbal IQ	105 (10.81)	100 (9.75)	F_(16)_ = 0.002, p = 0.32
Performance IQ	107.3 (12.07)	107.25 (11.7)	F_(16)_ = 0.06, p = 0.99
• CANTAB – Stockings of Cambridge			
Planning time (ms) ^a^	14084.8 (10912.79)	10135.16 (7703.62)	F_(16)_ = 2.01, p = 0.4
Execution time (ms) ^b^	1366.25 (2169.7)	2074.28 (2134.27)	F_(16)_ = 0.008, p = 0.5
Perfect solutions (number) ^c^	9.2 (2.74)	9.13 (1.13)	F_(16)_ = 4.81, p = 0.94
On-line task performance			
• *d′* prime			
1-back	3.12 (1.07)	3.5 (0.47)	Z = 33.5, p = 0.57
2-back	2.68 (1.28)	3.67 (0.17)	F_(16)_ = 2.18, p = 0.02
3-back	2.02 (0.83)	2.89 (0.83)	Z = 14, p = 0.02
• Reaction time (milliseconds)			
1-back	682.13 (258.98)	550.03 (154.43)	Z = 1.78, p = 0.83
2-back	625.99 (173.75)	570.26 (107.55)	F_(16)_ = 0.63, p = 0.44
3-back	707.53 (206.69)	659.11 (141.3)	F_(16)_ = 0.35, p = 0.58

+ Mean and standard deviation (SD) are presented, unless otherwise stated ^a^ The time taken to initiate the problems ^b^ The time taken to complete the problems ^c^ The number of problems solved in minimum moves ^a,b^ The planning and execution times were calculated at the highest level of difficulty (5 moves) to minimize possible ceiling effects.

### On-line task performance

There were no significant between-group differences in the mean number of correct responses given during the ‘easy’ and the ‘hard’ condition of the verbal fluency task (Table 1).

There were significant differences in the *d′* prime measure of on-line task performance between the PVH+VD and the UPVH groups during the 2- and 3-back conditions of the working memory task, but no significant differences in the mean reaction time during the n-back task (Table 2).

### fMRI results

Table 3 shows the between-group fMRI results.

**Table 3:** Between-group differences in regional brain activation with inreasing task difficulty during a verbal fluency and an n-back task.

Brain Region (Brodmann area)	Peak Talairach Coordinates (x, y, z)	Cluster size	Cluster p value
**Verbal fluency task (‘hard’≥‘easy’): VPT groups≥ controls**			
**R caudate nucleus extending to:**	**21, −30, 17**	**203**	**0.00027**
• Laterally - R insula (13) and superior temporal gyrus (BA 41)			
• Inferiorly - L caudate body and bilaterally to thalamus			
• Superiorly - R posterior cingulate gyrus (31), R precuneus (31), R superior temporal gyrus (39), R precentral gyrus (6) and R postcentral gyrus (2)			
**N-back task (3-back≥1-back): PVH+VD ≤UPVH**			
**L Inferior frontal gyrus (9) extends:**	**−40, 4, 26**	**192**	**0.00039**
• Anteriorly - L middle frontal gyrus (9) and L superior frontal gyrus (9)			
• Inferiorly - L middle frontal gyrus (46)			
• Superiorly - L precentral gyrus (6) and L middle frontal gyrus (8/6)			

R =  right; L = left.

#### Verbal fluency task

**Figure 1:**
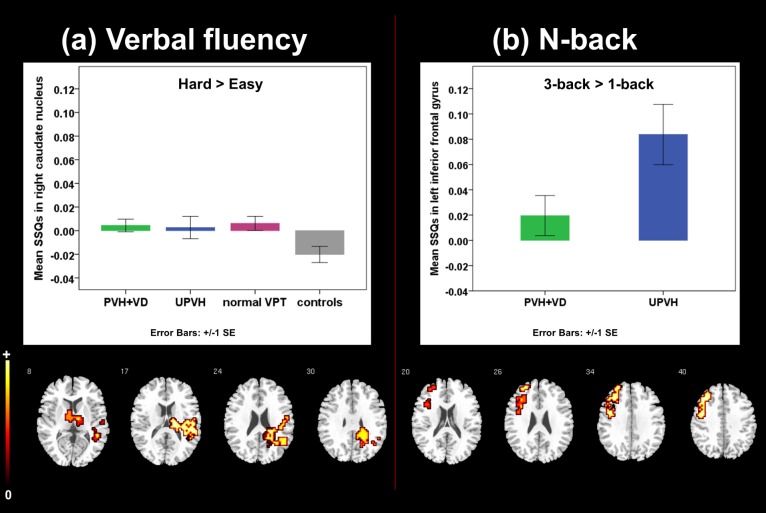
Between-group differences in regional brain activation as cognitive load increased during fMRI tasks. The numbers at the top of each row of slices represents the z coordinate in Talairach space. The right side of the brain corresponds to the right side of each slice.

There was a significant effect of group (i.e., VPT study groups>controls) as cognitive demands increased (‘hard’>‘easy’) in a cluster with local maxima in the right caudate nucleus. No significant effects of brain injury on BOLD signal were observed (Figure 1a).

#### N-back task

There were significant between-group differences (PVH+VD<UPVH) in the haemodynamic response to an increasing working memory load (3-back>1-back) in a cluster with local maxima in the left inferior frontal gyrus (Brodmann area-BA9) (Figure 1b).

To explore the relationship between the *d′* prime measure of on-line task performance, where between-group differences were observed during the 3-back condition, and SSQ values where significant interaction effects were noted, correlation analysis was carried out. Results showed that SSQ values in the left inferior frontal gyrus were not significantly associated with task performance (*r* = 0.43, p = 0.07).

## Discussion

### Verbal fluency

Our findings demonstrated that VPT-born young adults, regardless of extent of neonatal brain injury, exhibited a pattern of increased haemodynamic response to a phonological verbal fluency task as its cognitive demands increased (‘hard’>‘easy’) compared to term-born controls, in a large cluster that included the caudate nucleus, thalamus, insula, frontal, temporal and parietal cortices.

These results may partly be explained by structural alterations in the same brain regions that often accompany VPT birth [36], [53], [54], which are likely to exert some influence on activation patterns [41]. The lack of significant differences in caudate/thalamic activation between the three VPT samples grouped according to neonatal ultrasound classification is somewhat surprising, as alterations to these regions have been associated with cerebral haemorrhage [36], [55], [56]. Increasing cognitive load on a phonological verbal fluency task may have exceeded the capacities of the existing neural resources in the VPT group, resulting in the functional recruitment of additional neural resources [57]. Results from a previous fMRI investigation from our group using the same task, reported increased activation during ‘hard’ letters in the anterior cingulate gyrus and decreased activation during ‘easy’ letters in the same brain region in VPT young adults compared to controls [24]. Although different analyses methods were used in our previous study, these findings support the idea of increased recruitment of task-related brain regions with increased cognitive load. As task performance was similar in the four study groups, the between-group differences in BOLD signal that we observed here may not simply reflect differential task performance [58], although the strategies used by the four groups to complete the on-line task were not recorded and may indeed have differed.

Evidence from fMRI studies suggests that activation of the caudate nucleus, thalamus, insula and precentral and postcentral gyri is associated with articulatory demands [59]–[62] and that the superior temporal gyrus forms a part of the verbal fluency network sub-serving phonological aspects of the task [63]–[66]. Therefore, the greater engagement of these regions in the VPT groups seen here may have been necessary to achieve satisfactory on-line task performance. Increased activation of the caudate nucleus, precentral and postcental gyri has also been linked to increased cognitive demand, possibly relating to executive components of the verbal fluency task (15).

Furthermore, caudate nucleus has been involved in the suppression of irrelevant words, as well as of the activation of brain regions that may interfere with goal-oriented language production [67]–[69]. Therefore, the increased activation of the caudate nucleus in the VPT groups, as the cognitive demands of the task increased, may also reflect an increasing effort to maintain attention on task-related procedures and to suppress the generation of irrelevant words.

Differential activation across the study groups with increasing cognitive load was also observed in the right posterior cingulate gyrus (BA31) and right precuneus (BA31). Neuroimaging data have shown that the posterior cingulate and precuneus may play an important role in executive-type processes, such as response evaluation and monitoring, which are core cognitive components of verbal fluency processing [19], [20], [70]. Thus, the observed increased activation of the posterior cingulate and precuneus in the VPT groups may reflect a greater effort to avoid erroneous responses.

Taken together, the finding of altered activation in the VPT group during increasing cognitive load associated with phonological verbal fluency processing demonstrate long- term effects of preterm birth on the cortico-striatal-thalamo-cortical circuitry. These findings extend our previous research, which demonstrated altered activation in fronto-striatal pathways using the same task but considering ‘easy’ and ‘hard’ letter trials separately [24]. Alterations in the cortico-striatal-thalamo-cortical circuitry are likely to underlie the risk for specific motor, executive-type and emotional problems, which are part of the long-term sequelae of very preterm birth [71]–[73].

### Working memory

Our results showed that VPT young adults who sustained severe neonatal brain injury displayed decreased brain activation compared to VPT young adults with less-severe neonatal brain injury (UPVH) in left frontal brain regions in response to increasing working memory load of an n-back task (3-back>1-back). The cluster in which this significant interaction was observed was centred in the inferior/middle/superior frontal gyri (BA9), extending to the dorsolateral prefrontal cortex (DLPFC), which is a brain area thought to be centrally involved in working memory processing [74]. Hypoactivity in the DLPFC has been described in developmentally delayed populations, such as individuals with attention deficit hyperactivity disorder during executive-type tasks [75], [76]. A load-dependent role of the DLPFC in working memory maintenance has also been reported (15).

Between-group differences (PVH+VD<UPVH) that were linked to increasing task difficulty were also evident in the left precentral gyrus (BA6), and left middle frontal gyrus (BA6/8) i.e., supplementary motor cortex. Results from neuroimaging studies have shown that the precentral gyrus (BA6) and supplementary motor cortex may be involved in a sub-vocal rehearsal system of the phonological loop of working memory [29], [62], whereby maintenance of verbal information is achieved.

At a behavioural level, the PVH+VD group had lower *d′* scores than the UPVH group during the 3-back condition of the task, which is the most difficult, although there were no significant between-group differences in reaction time. The lack of significant association between *d′* scores achieved during the 3-back condition and functional data suggests that our fMRI results may not be solely attributable to on-line performance differences, and may instead reflect the long-term effects of neonatal brain injury.

Taken as a whole, decreased activation of frontal brain regions in the VPT group that suffered the most severe form of neonatal brain injury (PVH+VD), as task demand increased, may reflect limitations in neural resources [40], [57].

### Limitations

The fMRI data reported here may not generalise to VPT populations at large, as VPT individuals with compromised cognitive function were not studied. A further limitation of this study relating to the interpretation of on-line behavioural data arises from the fact that the study groups are relatively small for behavioural data analysis [77]. Finally, the population who undertook n-back task was small and included neither healthy controls nor VPT individuals with normal neonatal ultrasound results, which may prompt a caveat in the interpretation of the results.

## Conclusions

The results of the current study show that neonatal brain injury may exert differential effects on the functional neuroanatomy of various executive-type processes. Functional activation of cortico-striatal-thalamo-cortical circuitry associated with phonological verbal fluency appears to be increased following VPT birth, regardless of the extent of neonatal brain damage. This finding is in line with the results of other studies in VPT samples, which have documented the existence of functional neuroplastic adaptation in relation to language processing, reflected by the recruitment of additional task-related neural resources [24], [78]. On the other hand, the presence of severe neonatal brain injury may be associated with decreased engagement of the frontal cortex during verbal working memory processes, possibly because of its limited resource capacities due to maturational delays [28], [57], [79]. These data increase our understanding of the long-term consequences of early brain injury in ex-preterm individuals and may aid the development of neuro-protective treatments designed to improve long-term sequelae.
